# The Dual Role of Conjugated Linoleic Acid in Obesity and Metabolic Disorders

**DOI:** 10.1002/fsn3.70582

**Published:** 2025-07-22

**Authors:** Magendran Rajendran, Shanmugasundaram Palani, Thakur Gurjeet Singh, Brian Oliver, Kaml Dua, Vetriselvan Subramaniyan, Kumaran Narayanan

**Affiliations:** ^1^ School of Pharmaceutical Sciences Vels Institute of Science, Technology and Advanced Studies (VISTAS) Chennai Tamil Nadu India; ^2^ Chitkara College of Pharmacy Chitkara University Patiala Punjab India; ^3^ School of Life Sciences University of Technology Sydney City Campus Ultimo New South Wales Australia; ^4^ Discipline of Pharmacy, Graduate School of Health University of Technology Sydney Ultimo New South Wales Australia; ^5^ Department of Biomedical Sciences, Sir Jeffrey Cheah Sunway Medical School, Faculty of Medical and Life Sciences Sunway University, Malaysia Petaling Jaya Selangor Malaysia; ^6^ Jeffrey Cheah School of Medical and Health Sciences Monash University Subang Jaya Malaysia

**Keywords:** conjugated linoleic acid (cis‐9, trans‐11, CLA)/(trans‐10, cis‐12, CLA), insulin sensitivity, lipid metabolism, metabolic disorders, obesity

## Abstract

Conjugated Linoleic Acid (cis‐9, trans‐11, CLA)/(trans‐10, cis‐12, CLA) has been extensively studied for its role in obesity control and metabolic diseases. This review explores the molecular characteristics of CLA, its metabolic pathways, and its inconsistent effects on lipid metabolism, adipogenesis, energy expenditure, and inflammation. Preclinical and clinical studies suggest that CLA may promote fat oxidation and modulate adipocyte function; however, inconsistent findings highlight dose‐dependent outcomes and individual variability in response. The dual nature of CLA, showing both beneficial and adverse effects, raises questions about its long‐term safety and efficacy. This review critically examines CLA's molecular role in obesity and metabolic regulation, providing insights into its therapeutic promise and limitations. Future research should focus on personalized approaches to CLA supplementation, considering genetic and lifestyle factors for tailored nutritional guidance.

## Graphical abstract

Conjugated linoleic acid (CLA) modulates lipid metabolism, adipogenesis, energy expenditure, and inflammation, showing anti‐obesity effects in animal models. However, human studies reveal inconsistent outcomes due to dosage, isomer type, and individual variability. Personalized supplementation strategies are essential to maximize CLA's benefits while minimizing metabolic risks.

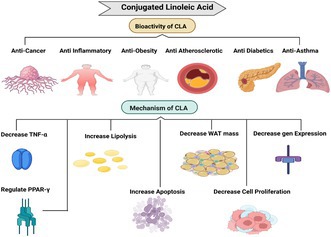

## 1 Introduction

One of the main public health issues is the rising rate of obesity, which affects individuals of all ages (Patias et al. 2024). The primary cause of the energy imbalance that results in the disease is overconsumption of high‐energy foods (Chowbey et al. 2022). In addition, metabolic syndrome has a close relation with oxidative stress, and hence the latter plays a role in diseases such as dyslipidaemia, diabetes, hypertension, musculoskeletal disorders, and cancer (Meyhöfer et al. 2022). There are several metabolic pathways it may employ to cause systemic oxidative stress, including the polyol and hexosamine pathways, oxidative phosphorylation, glyceraldehyde auto‐oxidation, and activation of protein kinase C (Manna and Jain 2015). The hormone TNF‐α and pro‐inflammatory adipokines like IL‐6, leptin, adiponectin, MCP‐1, and plasminogen activator inhibitor‐1 (PAI‐1) are differentially expressed in obese adipose tissue. Obesity pathogenesis can be implicated by these adipokines, either individually or in combination (Rocha and Folco 2011). The necessity for new strategies for the long‐term management of obesity is, therefore, paramount. In recent years, there has been a growing focus on complementary therapies and natural bioactive chemicals for the management of chronic diseases, especially in relation to inflammation and oxidative stress. Research has underscored the therapeutic potential of diverse natural compounds, including conjugated linoleic acid (CLA), royal jelly, French maritime pine bark extract, and *Momordica charantia* L., in alleviating inflammatory responses and oxidative damage linked to obesity and metabolic disorders (Lin et al. 2002; Bahari et al. 2023, 1995; Bhattacharya, Banu, et al. 2006). These findings endorse the incorporation of dietary interventions as supplementary measures in chronic illness management.

Ruminant diets virtually always include CLAs, a type of polyunsaturated fatty acid (Schmid et al. 2006). CLAs are conjugated isomers of octadecadienoic acid, most notably cis‐9, trans‐11 and trans‐10, cis‐12, derived from linoleic acid (C18:2n‐6, LA) through microbial biohydrogenation in the gastrointestinal tract of ruminant animals (Kennedy et al. 2010; Wallace et al. 2007). By changing the positions and structure of the double bonds, this process generates different CLA isomers, including trans‐10, cis‐12 and cis‐9, trans‐11 octadecadienoic acids. Commercial CLA products are synthesized from linoleic acid found in oils such as sunflower or safflower oil, typically under alkaline conditions. This process yields a CLA mixture that contains roughly equal parts of the 9,11 and 10,12 isomers (Pariza et al. 2001).

The cis‐9, trans‐11 isomer, also known as rumenic acid, comprises over 90% of CLA found in ruminant‐derived foods such as meat and milk, while the trans‐10, cis‐12 isomer accounts for the remaining 10%. Although multiple CLA isoforms exist, the 9,11 and 10,12 isomers exhibit the most significant physiological activities. In raw or processed beef and dairy products, CLA makes up approximately 0.34%–1.07% of total fat content (Silveira et al. 2007). First isolated by Ha et al. in 1987 (Ha et al. 1987), CLA has since garnered considerable attention due to the obesity epidemic over the past three decades. Numerous animal and human studies have demonstrated that CLA supplementation, particularly formulations containing both the 9,11 and 10,12 isomers or the isolated 10,12 isomer, can reduce body fat mass (Whigham et al. 2007; Wang and Jones 2004). The anti‐obesity effects of CLA have been largely attributed to the trans‐10, cis‐12 isomer, which has shown promising activity in both preclinical and clinical models (Park et al. 1999; Miller et al. 2008).

## 2 The Biochemistry of CLA

### 2.1 Structural Variants of CLA: c9, t11 Versus t10, c12 Isomers

**FIGURE 1:**
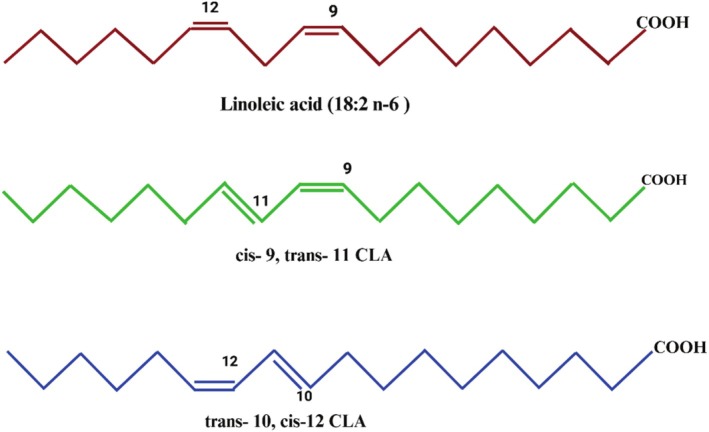
Structure of linoleic acid, *cis*‐9, *trans*‐11 CLA, and *trans*‐10, *cis*‐12 CLA.

CLA's double bonds are conjugated, in contrast to linoleic acid (LA), which has a methylene group separating its double bonds. This conjugation transpires when the double bonds are situated at carbon atoms 8–10, 9–11, 10–12, or 11–13, resulting in a conjugated diene configuration. The precise cis‐trans configuration of each isomer, including cis‐9, trans‐11 (c9,t11) and trans‐10, cis‐12 (t10,c12), dictates its unique physiological activity (Griinari et al. 1998). Figure 1 delineates the structural variations and distinctly identifies each isomer. There are two methods of classifying double bonds: cis and trans. While most of the CLA in foods is derived from the cis‐9, trans‐11 isomer, CLA‐rich foods also contain trace amounts of the trans‐10, trans‐12, trans‐9, trans‐11, and trans‐10, trans‐12 isomers. Figure 1 presents two isomers of CLA, cis‐9, trans‐11‐CLA and trans‐10, cis‐12‐CLA. Scientists have used CLA isomer mixtures primarily in their research to date, with each isomer making up 40%–45% of the total. Ruminant rumen bacteria biohydrogenate linolenic and linoleic acids to form the cis‐9, trans‐11 isomer (Roche et al. 2001). *Butyrivibrio fibrisolvens* , however, is a Gram‐negative bacterium that isomerizes LA to CLA. One of the methods of producing vaccenic acid or trans‐11‐octadecenoic acid is by engrossing or biohydrogenating the cis‐9, trans‐11 isomer (Kepler et al. 1996; Rodríguez Hernáez et al. 2018). Biohydrogenation or uptake of the cis‐9, trans‐11 isomer results in vaccenic acid or trans‐11‐octadecenoic acid. Once vaccenic acid is absorbed, it may be desaturated to cis‐9, trans‐11‐CLA by ∆9 desaturase. The most prevalent CLA in lactating cows' milk fat is cis‐9, trans‐11‐CLA, a derivative of trans‐11 octadecenoic acid, as per existing research. The animal feed composition and rumen microbiome composition may affect the concentration and distribution of CLA isomers in milk and beef, as per a recent study (MacDonald 2000).

### 2.2 Metabolic Pathways Influenced by CLA

**FIGURE 2:**
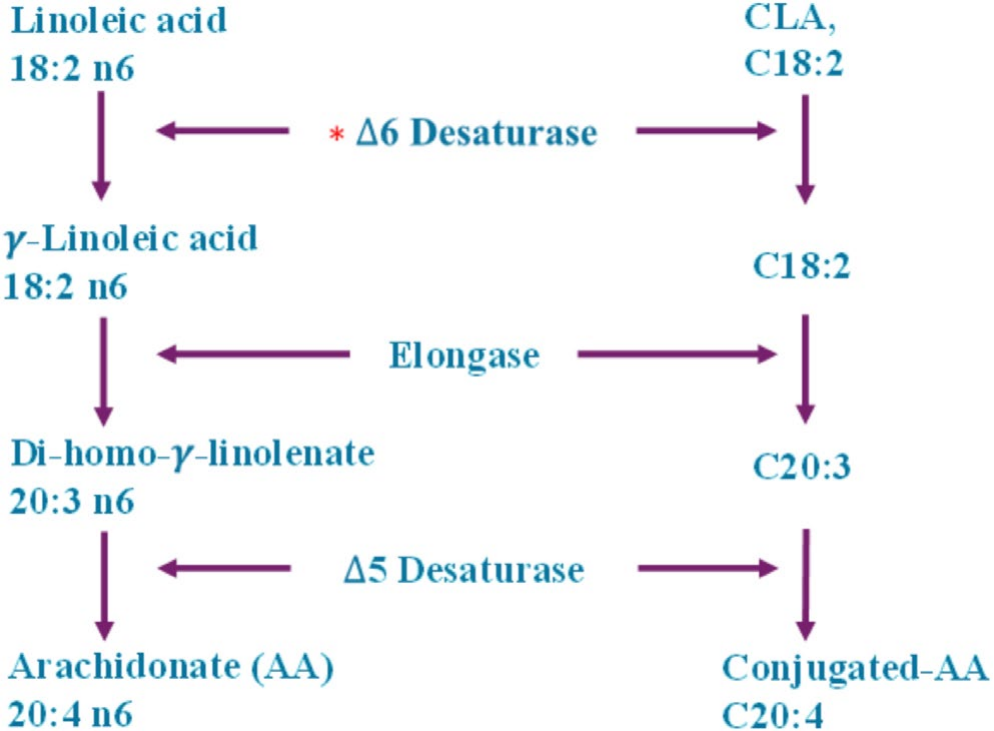
Metabolic pathway of conjugated linoleic acid.

**FIGURE 3:**
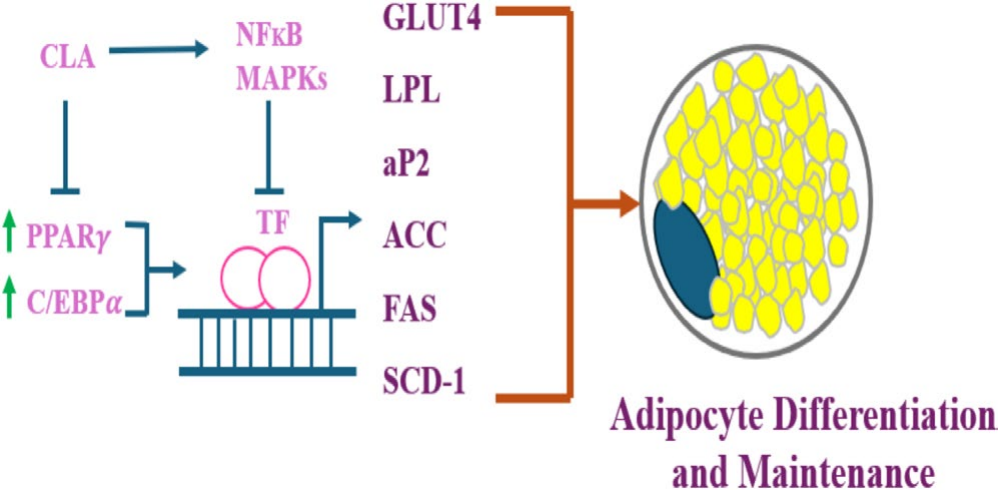
CLA inhibits adipogenesis by downregulating PPARγ and C/EBPα.

**FIGURE 4:**
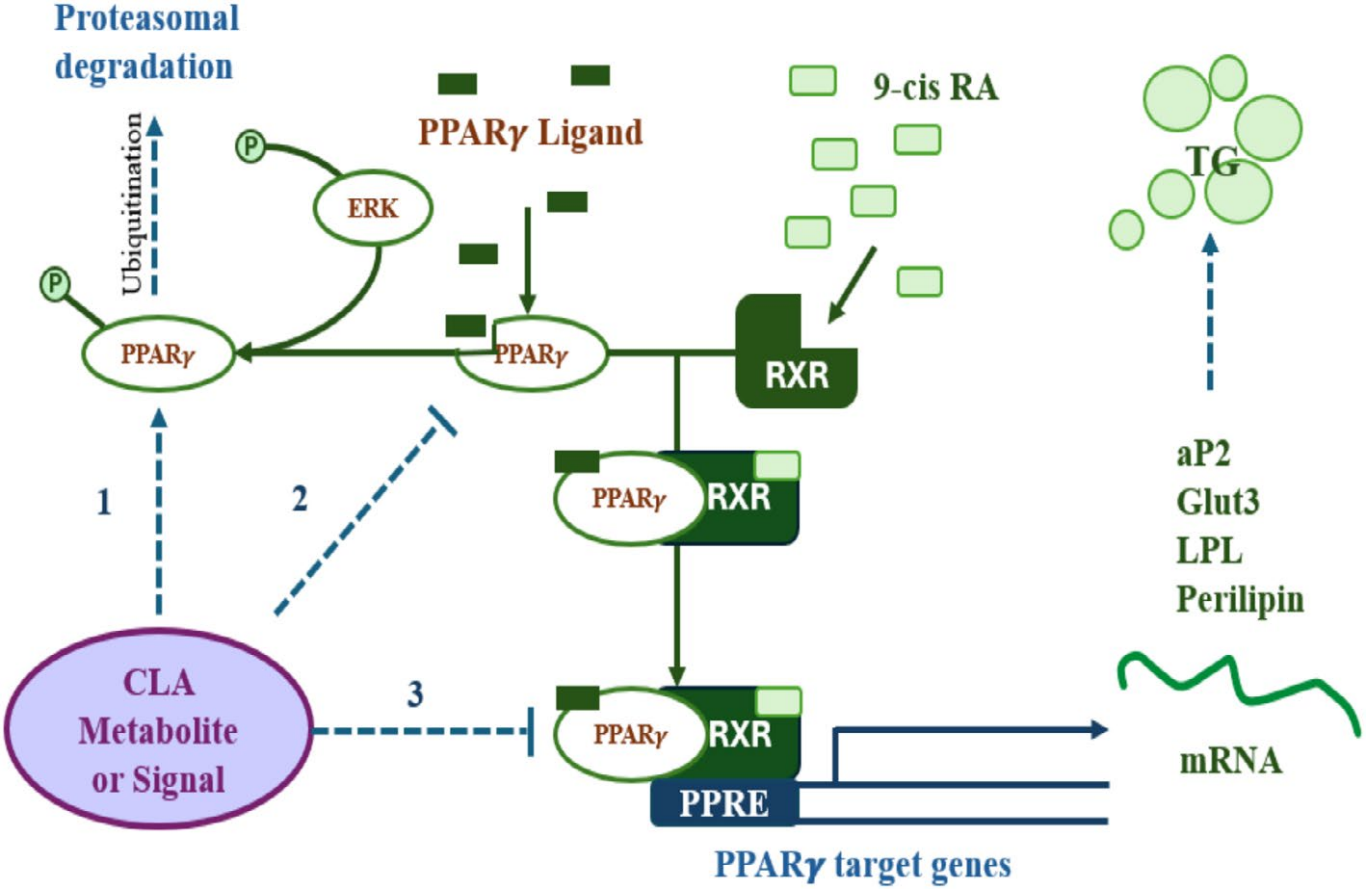
CLA reduces lipogenesis by downregulating key lipogenic enzymes.

CLA isomers are swiftly processed in the human body via various metabolic paths (Iversen et al. 1984). Extended and desaturated CLA metabolites (e.g., conjugated‐18:3, conjugated‐20:3, and conjugated‐20:4) have been recognized in rat liver and mammary tissue, as well as in human adipose tissue and serum (Ip 1997; Banni et al. 1995). The ∆6 desaturase enzyme in hepatic cells effectively metabolizes CLA (designated as C‐CLA) into C‐conjugated‐18:3, analogous to linoleic acid. CLA is converted into desaturation and elongation derivatives, as well as undergoing β‐oxidation in peroxisomes. This mechanism transforms elongated and desaturated CLA metabolites into smaller products such as 16:1 and 16:2 fatty acids (Belury and Kempa‐Steczko 1997; Banni et al. 1995). The importance of CLA metabolites in affecting tissue responses, including fat storage, glucose sensitivity, and cancer, remains under investigation; however, research is constrained by a lack of pure metabolites. To examine this, researchers administered conjugated nonadecadienoate (19:2), a 19‐carbon fatty acid believed to mimic CLA metabolites, to mice. Mice consuming a 19:2 diet exhibited an 81% decrease in fat accumulation, in contrast to a 25% decrease observed in mice administered CLA. Both 19:2 and CLA decreased lipid accretion and lipoprotein lipase action in 3 T3‐L1 preadipocytes, suggesting a potential involvement in fat metabolism (Brown, Evans, and McIntosh 2001; Park and Pariza 2001) (Figures 2, 3, 4, 5).

## 3 CLA and Obesity: Mechanistic Insights

### 3.1 Effects on Lipid Metabolism and Fat Oxidation

CLA lowers obesity via regulating lipid metabolism. It promotes fatty acid oxidation and modifies adipocyte glucose absorption, potentially affecting insulin sensitivity and inflammatory markers (Martins et al. 2015; Poirier et al. 2006). CLA participates in various physiological processes of fatty acids and is essential in lipid metabolism, especially in oxidative cellular conditions (Gudmundsen et al. 2000). Drastic alterations in lipid metabolism and fat shape have been caused by the numerous studies on the impact of CLA on energy metabolism (Kloss et al. 2005; Gaullier et al. 2005). Isoforms PPARα, PPARβ, and PPARγ constitute the nuclear receptor. PPARα plays a vital function in adipocyte formation (Abduljabbar et al. 2015; Tavares et al. 2007), but PPARα and β have roles in lipid and glucose metabolism. PPARγ controls the expression of genes for lipid metabolism in adipocytes, such as lipoprotein lipase and acyl CoA synthetase. PPARγ governs the synthesis of fatty acid transport proteins, which facilitate the uptake of these molecules by adipocytes. Metabolic alterations that inhibit lipogenesis and enhance lipolysis are one way CLA may change body shape. Another is skeletal muscle fatty acid oxidation. The enhanced activity of carnitine palmitoyltransferase‐1 or perhaps the inhibition of adipocyte growth is probably to blame (Churruca et al. 2009). Researchers examined whether CLA supplementation influenced hormone and lipid profiles and the metabolism of oxidation‐related enzymes (McGowan et al. 2013). A decrease in the adipocyte size, as opposed to their number, decreases body fat. Adipocyte size is directly proportional to their triglyceride content, and therefore a decrease in the latter results in a decrease in the former. By increasing the β‐oxidation of mitochondrial fatty acids, CLA can decrease the production of triacylglycerol, leading to smaller adipocytes instead of deposits (Botelho et al. 2005). Linoleic acid (LA, n‐6 PUFA) is converted to arachidonic acid, which leads to the production of pro‐inflammatory eicosanoids, but CLA is subject to β‐oxidation and isomerization, resulting in mixed metabolic and anti‐inflammatory effects. Conversely, n‐3 PUFAs, including EPA and DHA, are transformed into anti‐inflammatory mediators including resolvins and protectins. In comparison to n‐6 and CLA, n‐3 PUFAs more reliably promote anti‐inflammatory and cardioprotective effects. The fatty acid is brought into the mitochondria by the carnitine‐palmitoyltransferase (CPT) complex. CPT‐1 and CPT‐2 enzymes and carnitine acylcarnitine translocase (CATC) are all that are needed. By binding with the carnitine‐palmitoyltransferase (CPT‐1) enzyme, the acyl‐CoA synthetase enzyme forms the active compound fatty acyl‐CoA. This complicated process crosses through the intermembrane space following a passage through the mitochondrial membrane. Releasing carnitine through the CPT‐2 pathway refills acyl‐CoA. The LCFA generates adenosine triphosphate (ATP) through β‐oxidation after it enters into the mitochondrial matrix (Holloway et al. 2006).

The activity and concentration of CPT‐1, an enzyme that facilitates the transport of fatty acids into the mitochondria for energy production, can be enhanced by the intake of CLA supplements. Fatty acid storage in fat and muscle tissues is reduced, and lipolysis is enhanced due to this. The activity of lipoprotein lipase (LPL) is also decreased by CLA, which contributes to fat loss. The Krebs cycle (CK) is where fatty acids undergo β‐oxidation in the mitochondria, generating electrons (e‐) and hydrogen ions (H+). These are passed on to the respiratory chain through NADH and FADH2. This results in an electrochemical gradient which, when it passes through ATP synthase, leads to the generation of ATP. They can also break down proteins in order to generate heat (Lehnen et al. 2015). The impact of CLA on lipolysis by enhanced CPT‐1 activity is most apparent when γ‐oxidation, the process of breaking down fatty acids to synthesize ATP, performs better than the transport of fatty acids into the mitochondria. Thus, the most probable group to gain the most from consuming CLA would be those who exercise frequently, particularly those whose β‐oxidation is superior to transporting fatty acids to the mitochondria (Lehnen et al. 2015).

### 3.2 CLA and Obesity: Mechanistic Insights Modulation of Adipogenesis and Lipogenesis

Two crucial transcription factors participating in the differentiation of preadipocytes into adipocytes are peroxisome proliferator‐activated receptor (PPAR) γ and CAAT/enhancer binding proteins (C/EBPs). PPAR γ and C/EBP α, the key controllers of adipocyte development, become activated during differentiation due to increased activity of C/EBP β and C/EBP δ. It has been demonstrated that CLA therapy effectively reduces adipogenesis and lipogenesis by reducing expression of PPAR γ, C/EBP α, SREBP‐1c, LXR α, and aP2. In rodents, the target gene and PPAR γ expression were lower when supplemented with 10,12 CLA. Extensive literature reveals that CLA suppresses the formation of preadipocytes in animals and hominids (Lehnen et al. 2015; Evans et al. 2000; Kang et al. 2003; Brown et al. 2003, 2004). Further evidence reveals that 10,12 CLA is capable of reducing and even reversing the process of adipogenesis, which is likely made possible through modulation of PPAR γ activity. Reduced expression of the PPAR γ target gene may be the result of lowered PPAR γ expression or post‐translational inhibition of its activity. Because PPAR γ regulates its own expression, reducing its activity would also reduce its expression, making it more difficult to determine whether the inhibition occurs at the transcriptional or post‐translational phase (Brown et al. 2004) of examination. Moreover, when CLA provokes inflammation in adipocytes, it may hinder PPAR γ activation. 10,12 CLA activates adipocytes via NF κB, therefore increasing the expression of proinflammatory cytokines (Chung, Brown, Provo, et al. 2005).

Accumulation of triglycerides is the main function of adipocytes. Various proteins involved in lipogenesis, such as stearoyl‐CoA desaturase (SCD), acetyl‐CoA carboxylase (ACC), fatty acid synthase (FAS), and lipoprotein lipase (LPL), have been reported to be decreased upon supplementation with various isomers of CLA or 10,12 CLA individually (Lin et al. 2002; LaRosa et al. 2006). Several lipogenic genes like glycerol‐3‐phosphate dehydrogenase (GPDH), lipin, and lipoprotein lipase (LPL) are substantially stimulated when PPARγ is active. There is also up‐regulation of many genes that encode for proteins associated with lipid droplets such as perilipin, ADRP, CIDEC, and S3‐12. Some of the fault may be attributable to CLA's anti‐lipogenic activity, achieved by inhibiting activation of PPARγ. The lipogenic transcription factor SREBP‐1 and the target genes thereof can also be greatly affected by CLA prevention. Finally, insulin‐dependent glucose transporter 4 (GLUT4), LPL, ACC, FAS, and SCD‐1 can have their activation or predominance affected by CLA inhibition of insulin action (Nielsen et al. 2008).

In rat and human adipocyte culture, CLA combinations decreased the content of monounsaturated fatty acids (Martin et al. 2000). Their ability to inhibit SCD1 expression (Choi et al. 2000) and activity, implicated in monounsaturated fatty acid synthesis, is most likely responsible. Of interest, even in SCD1 knockout mice, CLA reduced body weight and changed the fatty acid composition (increasing 16:0/16:1 and decreasing 18:0/18:1). Other desaturases, such as SCD‐2 or Δ6‐desaturase, may be involved in the anti‐obesity effect of CLA, considering the association between Δ6‐desaturase activity and decreased body fat in CLA‐treated mice (Hargrave‐Barnes et al. 2008).

### 3.3 Role in Energy Expenditure and Thermogenesis

The correlation between energy use and expenditure dictates energy equilibrium. Body weight and body fat mass upsurge when energy consumption surpasses energy expenditure and reduction when the opposite occurs. Consequently, plummeting energy consumption or accumulative energy expenditure are two potential methods by which CLA reduces body fat mass (Park et al. 1997). CLA enhances energy expenditure. Adaptive thermogenesis, physical activity, and basal metabolic rate (BMR) all affect energy expenditure. CLA is projected to decrease obesity by augmenting energy spending via elevated BMR, thermogenesis, or lipid oxidation in animals (Nagao et al. 2003; West et al. 2000). Mixed CLA isomers elevated the basal metabolic rate and decreased body fat by 50% in male BALB/c mice following 6 weeks of administration (Terpstra et al. 2003). CLA supplementation may correlate with enhanced thermogenesis due to higher levels of uncoupling proteins (UCPs), which promote proton transport across the inner mitochondrial membrane, hence redirecting energy utilization from ATP synthesis to heat generation. The predominant UCP2 is uttered in various tissues, comprising white adipose tissue (WAT) (House et al. 2005; Takahashi et al. 2002; Ealey et al. 2002). UCP1 is predominantly located in brown adipose tissue, whereas UCP3 is primarily present in muscle and other tissues. While its role in energy dissipation remains uncertain, it has been shown that CLA (LaRosa et al. 2006), particularly 10,12 CLA, enhances UCP2 expression in white adipose tissue (WAT). Moreover, CLA enhances the appearance of CPT1 in white adipose tissue, hence facilitating the uptake of mitochondrial fatty acids and augmenting beta‐oxidation. These effects have been observed in rodent muscle, liver, and developing preadipocytes (Ferramosca et al. 2008; Roche et al. 2002; Rahman et al. 2007).

Weight loss and elevated energy expenditure were demonstrated in a single trial. Subjects given a CLA combination supplement for 6 months at 4 g per day lost weight and exhibited evidence of enhanced energy expenditure and sleep oxidation, based on research conducted by Close et al. (LaRosa et al. 2006). It is thought that by enhancing osteogenic gene expression and inhibiting osteoclast activity, CLA supplements may enhance bone mineral density. When middle‐aged female mice were supplemented with CLA or exercised with them, their bone mineral density was enhanced, which was similar to the control group. In contrast, 9,11 CLA enhanced adipocyte differentiation while suppressing osteoblast differentiation. Rats treated with a mixture of CLA and corticosteroids (which inhibit muscle and bone mass) exhibited a reduction in LBM, bone mineral content, and bone mineral density (Roy D et al. 2008), consistent with our in vitro findings.

## 4 Clinical Outcomes of CLA in Obesity Research

### 4.1 Human Trials: Weight Loss and Body Composition

Evaluating CLA investigations in human trials is problematic due to restricted sample sizes, varying CLA doses and isomers, diverse treatment durations, and differing demographic characteristics of the research subjects. Recent findings from several human studies have led to CLA being granted Generally Recognized as Safe (GRAS) status in the United States. No significant adverse effects were recorded in clinical research; however, modest gastrointestinal disturbances were observed in a small cohort of persons (Risérus et al. 2002; Moloney et al. 2004). Concise experiments with single‐isomer supplementation revealed an elevation in insulin resistance and a reduction in HDL cholesterol levels (Song et al. 2005). Dale Schoeller conducted a study yielding similar results, concentrating on the effectiveness of CLA in mitigating weight gain on vacations. Forty healthy obese men volunteered for this double‐blind, placebo‐controlled, randomized study between August and March and received either a placebo or 3.2 g/day mixed CLA. Results indicated that CLA reduced body fat more so than placebo without adversely affecting inflammation, insulin resistance, or liver function (Watras et al. 2007). Individuals who were overweight and had early signs of metabolic syndrome (MetS) (i.e., at least two of the following: high blood pressure, fasting glucose, triglycerides, or low HDL cholesterol) were found to have reduced fat when they received 3 g of mixed CLA per day. There were no adverse effects on glucose or liver metabolism. The outcomes of this study concur with those of another finding that CLA supplementation is well tolerated overall. One individual resigned from CLA due to gastrointestinal distress, perhaps exacerbated by a simultaneous influenza infection. Notably, akin to the sole previous CLA trial involving children, this investigation revealed no significant alterations in fasting glucose, insulin levels, or insulin resistance, suggesting that CLA exerted no adverse effects on these metabolic parameters within this cohort (Racine et al. 2010).

Numerous research studies, including those in Table 1, have shown that CLA supplementation positively influences body mass and adiposity‐related measures, with no indication of negative effects (Tables 2 and 3).

**TABLE 1:** A summary of research on human CLA supplementation.

CLA dosage and groups	Study design	Participants	Participants	References
Mixed CLA: 3.2 g/day (*n* = 20), 6.4 g/day (*n* = 18); Placebo (*n* = 17)	12‐week randomized, double‐blind trial	Healthy adults (*n* = 55)	Increased lean body mass; elevated serum CRP and IL‐6	(Steck et al. 2007)
Mixed CLA (1.7–6.8 g/day); Placebo (9 g olive oil/day)	12‐week randomized, double‐blind trial	Overweight adults (*n* = 52)	Reduced fat mass (< 3.4 g/day); increased lean mass (< 3.4 g/day)	(Gudmundsen et al. 2000)
9,11 CLA (3 g/day) vs. placebo (olive oil)	12‐week randomized, double‐blind trial	Obese men (*n* = 25)	Worsened insulin resistance; increased fat mass	(Risérus et al. 2004)
Mixed CLA (5.5 g/day), 9,11 CLA (4.7 g/day), or placebo	16‐week randomized, double‐blind trial	Postmenopausal women (*n* = 81)	Reduced total and lower‐body fat	(Raff et al. 2009)
Mixed CLA (4.2 g/day) vs. placebo	4‐week randomized, double‐blind trial	Abdominally obese older men (*n* = 25)	Decreased sagittal abdominal diameter	(Risérus et al. 2001)
Microencapsulated CLA (3 g/day) + low‐calorie diet vs. placebo	90‐day randomized trial	Women with metabolic syndrome (*n* = 14)	Lowered insulin levels; reduced body fat; no lipid changes	(Carvalho et al. 2012)
Mixed CLA (3 g/day), metformin (1 g/day), or placebo	16‐week randomized, double‐blind trial	Obese children (*n* = 50, ages 8–18)	Improved insulin sensitivity	(Garibay‐Nieto et al. 2017)
Mixed CLA (5 g/day, *n* = 38) vs. placebo (*n* = 38)	14‐week randomized, double‐blind crossover trial during resistance training	Healthy adults (*n* = 76)	Increased muscle mass; reduced fat mass and percentage; enhanced strength	(Pinkoski et al. 2006)
Mixed CLA (1.8 or 3.6 g/day) vs. placebo (oleic acid)	13‐week randomized, double‐blind trial	Overweight adults (*n* = 54)	No weight regain difference; reduced appetite; increased satiety	(Kamphuis et al. 2003)

**TABLE 2:** Effects of CLA on obesity and metabolism.

Purpose of the study	Key findings	Diagnosis/model	Implications	References
Anti‐obesity effects of CLA	CLA showed strong anti‐obesity effects in animals but only moderate results in humans	Animal and human studies	CLA may aid weight management in animals, but human efficacy needs more research	(Park and Pariza 2001; Onakpoya et al. 2012)
Impact of CLA on BMI, weight, and body composition	CLA supplementation improved BMI, body fat, abdominal adiposity, and lean mass	Obesity‐related parameters	CLA could be a beneficial supplement for obesity management and body composition	(Zeng et al. 2024)
Impact of CLA on BMI, weight, and body composition	CLA supplementation improved BMI, body fat, abdominal adiposity, and lean mass	Obesity‐related parameters	CLA could be a beneficial supplement for obesity management and body composition	(Zeng et al. 2024)
Low‐dose CLA (100 mg/kg/day) on metabolic health	CLA counteracted metabolic disruptions caused by a CAF diet, enhancing metabolic health	Diet‐induced obesity model	Low‐dose CLA may help improve metabolic health in obesity without major side effects	(Martin et al. 2000)
CLA effects in mice with paternal Snord116 deletion	CLA reduced body weight and fat in Snord116‐deficient mice on a high‐fat diet	Prader‐Willi Syndrome (PWS) model	CLA might help manage obesity in PWS, but further studies are required	(Knott et al. 2022)
Combined CLA and chromium (Cr) effects on obesity	CLA + Cr reduced weight, fat mass, and inflammation while improving insulin sensitivity	Obesity and metabolic dysfunction model	CLA and Cr together could enhance obesity treatment and metabolic health	(Banu et al. 2008)
CLA‐induced apoptosis in adipose tissue	CLA triggered fat cell death in white adipose tissue but did not increase energy expenditure	Adipose tissue analysis	CLA may reduce fat by promoting apoptosis, offering a unique weight‐loss mechanism	(Miner et al. 2001)
Dose‐dependent CLA effects on body fat and energy intake	Lower CLA doses (0.5%–1.0%) reduced fat (especially retroperitoneal fat) without affecting food intake	Body composition analysis	Even low CLA doses may support fat loss and muscle preservation without dietary restrictions	(DeLany et al. 1999)

**TABLE 3:** Comprehensive mechanisms and effects of conjugated linoleic acid (CLA).

Category	Mechanism	Key outcomes	References
Anti‐obesity	Stimulates fatty acid β‐oxidation	Reduces adipocyte size in mice (not quantified in humans)	(Tsuboyama‐Kasaoka et al. 2000)
	Downregulates PPARγ in 3 T3‐L1 adipocytes	Decreases BMI in meta‐analysis (0.5 kg/m^2^)	(Derakhshande‐Rishehri et al. 2015)
	Activates AMPK in mice (not quantified as %)	Maintains lean mass in rodents	(Jiang et al. 2009)
Anti‐inflammatory	Reduces TNF‐α production in bovine cells	Lowers CRP in humans (0.8 mg/L)	(Perdomo et al. 2011), (Penedo et al. 2013)
	Increases IL‐10 in cell studies (no fold‐change reported)	Improves endothelial function (clinical correlation)	(Valenzuela et al. 2023)
Cardiometabolic	Inhibits hepatic SCD‐1 mRNA (70% reduction in rats)	Lowers LDL in hamsters (not quantified in humans)	(Lee et al. 1998), (Nicolosi et al. 1997)
Anti‐cancer	Induces apoptosis in mammary tumors (no caspase‐3 quantification)	Reduces tumor incidence by 50% in rats	(Białek et al. 2015)
	Inhibits ovarian cancer growth (70% reduction)	Shows isomer‐specific effects	(Thuillier et al. 2013)
Neuroprotective	Activates PPARα in microglia (no % reduction reported)	Reduces neuroinflammation markers	(Murru et al. 2021)

### 4.2 Evidence From Animal Models: Translational Significance

Since its initial study in 1997, the anti‐obesity benefits of CLA have captivated researchers' interest. Nonetheless, as van Hartigh indicates, the comprehensive results from human studies are moderate in contrast to the more pronounced effects observed in animal studies. Nonetheless, numerous clinical trials have demonstrated that CLA supplementation enhances BMI, weight, body fat mass (BFM), abdominal adiposity, and lean body mass (LBM) (Bhattacharya, Rahman, et al. 2006; Zeng et al. 2024). CLA augmented total energy expenditure in animal prototypes, but not in hominids (Chin et al. 1994). Low‐dose CLA supplementation, specifically at 100 mg/kg/day, was shown by Miguel Z. Martín‐González to successfully alleviate metabolic disturbances caused by a CAF diet, enhancing obesity‐related metrics and metabolic health without notable side effects (Martín‐González et al. 2020). A separate study reveals that mice with a paternal deletion of Snord116 (Snord116m+/p−) and those with a homozygous deletion of Snord116 alleles have reductions in body mass and fat when subjected to a high‐fat/CLA diet, signifying that the Snord116 genotype does not impede the weight‐reducing effects of CLA. The findings indicate that CLA could function as a possible dietary supplement for obesity management in persons with Prader–Willi Syndrome (PWS), necessitating additional mechanistic and translational study (Knott et al. 2022). A study examining the synergistic effects of CLA and chromium (Cr) revealed that this combination significantly decreases body mass, total physique fat, and visceral fat mass in obese mice produced by a high‐fat diet, providing superior advantages paralleled to CLA alone. This integrated therapy enhanced energy expenditure, oxygen uptake, and insulin sensitivity while decreasing serum leptin, pro‐inflammatory cytokines, and the insulin resistance index. These results underscore the potential of CLA and Cr as a therapeutic approach for addressing obesity and metabolic dysfunction (Bhattacharya, Rahman, et al. 2006). Another study indicated that dietary CLA promotes apoptosis in white adipose tissue within 5 days of consumption, as evidenced by increased DNA fragmentation in retroperitoneal fat pads. CLA diminishes feed intake by 10%–12% and reduces the weights of white and brown adipose tissues; however, it does not markedly enhance energy expenditure. These findings underscore CLA's possible function in diminishing adipose tissue via apoptosis rather than modifying metabolic energy consumption (Miner et al. 2001).

The research reveals that CLA significantly diminishes body fat buildup in mice without inhibiting food consumption. At dosages of 0.50%, 0.75%, and 1.0%, CLA markedly reduced body fat, especially in the retroperitoneal fat depot, while enhancing carcass protein content. These benefits were noted at 2 weeks and continued for 12 weeks of CLA administration. The results indicate that CLA facilitates fat reduction and protein enhancement in body composition at very modest dosages, irrespective of its effect on food consumption (DeLany et al. 1999).

### 4.3 Safety and Adverse Effects: A Dual‐Edged Sword

The safety and efficacy of CLA have been well examined concerning obesity and metabolic syndrome. A study indicated that daily supplementation with 3.4 g of a CLA isomer blend (50:50 c9,t11:t10,c12) is safe for healthy overweight or obese persons, showing no significant differences in adverse events or safety metrics between the CLA and placebo groups. A little decrease in body weight and BMI was noted, although the overall impact on body composition was negligible. The data indicate that while CLA isomer blends are safe, their effectiveness for weight loss is constrained, highlighting the necessity for more dose–response studies (Behr et al. 2000). A separate trial indicated that CLA supplementation (3 g/day) for 12 weeks in overweight and obese women was well tolerated and did not adversely affect liver function. The supplementation led to substantial decreases in total body fat, encompassing reductions in android, gynoid, and visceral adipose tissue, along with augmentation in lean body mass. Significantly, there were no notable alterations in liver enzyme activity, signifying the nonappearance of liver‐related adverse possessions. The data indicate that CLA supplementation may enhance body fat conformation in overweight and obese females (Mądry et al. 2020).

A distinct study investigating the impact of CLA supplementation on bone health in overweight and obese females revealed no notable enhancements in bone mineral density (BMD) or bone mineral content (BMC) after a duration of 3 months. Despite a notable enhancement in BMC and BMD at the lumbar spine within the CLA group, no disparities were observed among the CLA and placebo groups regarding the other bone measures. The results designate that CLA supplementation does not offer noteworthy advantages for bone health, and extended research is required to thoroughly evaluate its potential effects (Jamka et al. 2023).

#### 4.3.1 Influence of CLA on Insulin Resistance

CLA is a lipid present in specific dietary supplements. It is frequently advocated for weight loss, although it may not effectively assist individuals in losing weight and could even jeopardize their health. Although certain forms of CLA may confer health benefits, a specific variant known as t10, c12 CLA could pose risks. It may induce problems including lipodystrophy, insulin resistance, and reduced milk production in lactating mothers. Individuals should refrain from using CLA supplements until further research substantiates their safety and efficacy (Larsen et al. 2003). In human research, the prolonged use of CLA supplements necessitates the monitoring of insulin, glucose levels, and HDL (a specific form of cholesterol). CLA has documented negative effects, including gastrointestinal issues and exhaustion (Gudmundsen et al. 2000). A significant concern identified in human investigations is that CLA primarily influences the potential promotion of colon cancer through its effects on pathways associated with NF‐kB and cyclin D1 (Rajakangas et al. 2003). CLA has been related to advantageous health properties; nevertheless, potential adverse implications cannot be dismissed. The adverse effects encompass gastrointestinal complications, fatty liver disease, splenic disorders, augmented risk of colon cancer, and hyperproinsulinaemia. The safety of CLA for humans is still uncertain, as variables such as the type of CLA, dosage, formulation, duration, and individual characteristics (e.g., age, gender, ethnicity) require additional investigation. Nonetheless, research indicates that CLA may operate as a functional meal with comprehensive health advantages (Pariza 2004; Cheng et al. 2003; Kramer et al. 2004).

## 5 CLA in the Context of Metabolic Disorders

### 5.1 Mechanisms of Insulin Sensitivity and Resistance

CLA is linked to insulin resistance, and some mixtures of CLA, such as the c9,t11 form, may be advantageous for regulating insulin resistance. CLA has been documented to possess the capacity to diminish body fat in both human and animal research. It predominantly regulates adipose tissue by modulating the gene expression of enzymes associated with fatty acid production, uptake, and triglyceride synthesis. Although distinct species employ varied lipogenic organs, including the liver and adipose tissue, analogous pathways for lipid production are present in these tissues. CLA supplementation has been demonstrated to affect and diminish fat accumulation in various tissues (House et al. 2005; Kramer et al. 2004). Other studies have associated the c9,t11‐isomer with decreased blood lipids in ob/ob mice and improved insulin sensitivity via diminished TNF‐α expression in adipose tissue. The divergent reactions seen in normal versus insulin‐resistant animals underscore the necessity for more study to explain the influence of certain CLA isomers on metabolic processes and the development of metabolic syndrome (Taylor and Zahradka 2004). As previously shown, CLA did not influence gene expression in adipose tissue. Nonetheless, the administration of CLA decreased mRNA levels and LPL. LPL catalyzes the hydrolysis of extracellular triglycerides, liberating free fatty acids for cellular absorption. CLA showed more efficacy in diminishing LPL gene expression and activity in adipocytes (Zabala et al. 2006; Park et al. 2004). CLA can experience restricted desaturation and elongation in extrahepatic tissues such as the testes and kidneys. Nonetheless, the liver serves as the principal location for fatty acid metabolism, whereas other organs display diminished enzymatic activity.

### 5.2 The Role of CLA in Inflammation and Adipokine Regulation

CLA has been projected as a viable method for formulating novel and safer nutritional therapies to address inflammation. Given that inflammation is a significant contributor to numerous prevalent diseases comprising inflammatory bowel disease (IBD), arthritis, atherosclerosis, obesity, diabetes, asthma, infections, and cancer, CLA offers a prospective alternative or adjunctive approach. It may effectively interrupt the inflammatory procedure deprived of inducing opposing side effects, rendering it a significant alternative for addressing inflammation‐related disorders (Zeng et al. 2024), (Xu et al. 1999). CLA may confer advantages for fatty liver disease by influencing lipid metabolism and diminishing hepatic fat buildup, as indicated by recent research. Nonetheless, its effects are contingent upon the isomer type, dosage, and individual metabolic circumstances (Zeng et al. 2024).

#### 5.2.1 CLA and Immune Response

Research in both animals and individuals has established that CLA exerts positive effects on immunological and inflammatory responses. It has been shown to diminish colonic inflammation, decrease cytokine production by immune cells, and mitigate opposing consequences of immunological challenges. CLA additionally affects inflammatory mediators such as cytokines, prostaglandins, leukotrienes, and immunoglobulins (Oleszczuk et al. 2012). Both principal CLA isomers (c‐9,t‐11 and t‐10,c‐12) modulate the immune system by diminishing the activity of essential immune cells, comprising monocytes, macrophages, dendritic cells, and natural killer cells. Furthermore, CLA can augment adaptive immune responses, rendering it especially advantageous for those with compromised immune systems. Supplementation with CLA has demonstrated an enhancement in antibody production and a reduction in inflammatory mediators across multiple investigations. It also contributes to the regulation of allergic responses and airway inflammation by altering essential immunological factors (Yamasaki et al. 1999).

#### 5.2.2 CLA as a Cytokine Expression Modulator

Studies indicate that CLA, chiefly the c‐9,t‐11 isomer, can reduce the amalgamation of pro‐inflammatory cytokines such as IL‐6, TNF‐α, IFN‐γ, and IL‐1β, which are associated with chronic inflammation‐related disorders (Plourde et al. 2008). Recent in vitro investigations utilizing bovine blood have demonstrated that the t‐10,c‐12 CLA isomer can reduce TNF‐α manufacture in immune cells activated by LPS (a bacterial toxin), while linoleic acid and c‐9,t‐11 CLA exhibited no meaningful impact. An ex vivo investigation on porcine immune cells showed that t‐10,c‐12 CLA decreased NF‐κB activation and TNF‐α mRNA levels upon exposure to endotoxins. These findings indicate that CLA, especially the t‐10,c‐12 isomer, contributes to the reduction of inflammation at the cellular level (Kennedy et al. 2010).

#### 5.2.3 CLA‐Mediated Anti‐Inflammatory Mechanisms via PPARγ

Peroxisome proliferator‐activated receptors (PPARs) are nuclear receptors functioning as transcription factors that regulate genes associated with energy homeostasis, glucose metabolism, and immune response. These receptors are prevalent in immune cells such as macrophages, dendritic cells, T cells, and epithelial cells, where they affect cellular proliferation, programmed cell death (apoptosis), and inflammation (Moya‐Camarena et al. 1999). There exist three categories of PPARs: PPARα, PPARδ/β, and PPARγ. CLA, namely its c‐9, t‐11; t‐9, t‐11; and t‐10, c‐12 isomers, serves as potent activators of PPARα and PPARβ, resulting in alterations in gene expression associated with metabolic and immunological responses. These isomers significantly activate PPARγ, which is crucial for mitigating inflammation by modulating cytokines, chemokines, and survival factors in immune cells (Krey et al. 1997).

#### 5.2.4 Regulation of CLA and Adipokines

The t10, c12‐CLA isomer is related to insulin resistance owing to its influence on adipokines and inflammatory pathways. It elevates the discharge of pro‐inflammatory cytokines such as IL‐6, IL‐8, MCP‐1, and TNF‐α, which disrupt glucose metabolism and lead to metabolic dysfunction. The effects are arbitrated by the initiation of JNK and ERK pathways, resulting in elevated calcium ion levels and oxidative stress (Chung, Brown, Sandberg, and McIntosh 2005; Jiang et al. 2009). Furthermore, t10, c12‐CLA diminishes adiponectin secretion, an anti‐inflammatory and insulin‐sensitizing adipokine, while c9, t11‐CLA enhances adiponectin synthesis and optimizes adipocyte function. t10, c12‐CLA also impedes adiponectin oligomerization, diminishing its biological efficacy, thereby exacerbating insulin resistance associated with obesity (Miller et al. 2008; Brown, Halvorsen, et al. 2001).

### 5.3 CLA and Cardiovascular Disease (CVD) Risk Factors

Cardiovascular diseases (CVD) remain to be a chief reason for mortality, prompting investigations into the possible advantages of CLA for cardiac health (Nicolosi et al. 1997; Li et al. 2023). Animal investigations propose that CLA might have anti‐atherosclerotic properties, reducing total cholesterol, triglycerides (TG), LDL cholesterol, and blood pressure while increasing HDL cholesterol (Lee et al. 1998). The suggested processes encompass PPARs, SREBPs, and SCD, which govern lipid metabolism (Kritchevsky 2000). Nonetheless, human research regarding the cardiovascular effects of CLA remains ambiguous. Some studies indicate beneficial effects on lipid profiles, whilst others demonstrate no change or possibly adverse effects, including a reduction in HDL cholesterol, a crucial protective lipid (Choi et al. 2000). The t10,c12‐CLA isomer is linked to elevated LDL/HDL ratios, potentially heightening cardiovascular disease risk, while c9,t11‐CLA seems to be more neutral or advantageous. Further investigation is required to ascertain the comprehensive properties of CLA on cardiovascular wellbeing (Risérus et al. 2004).

## 6 The Paradox of CLA

### 6.1 Contradictory Evidence: Balancing Benefits and Risks

**FIGURE 5:**
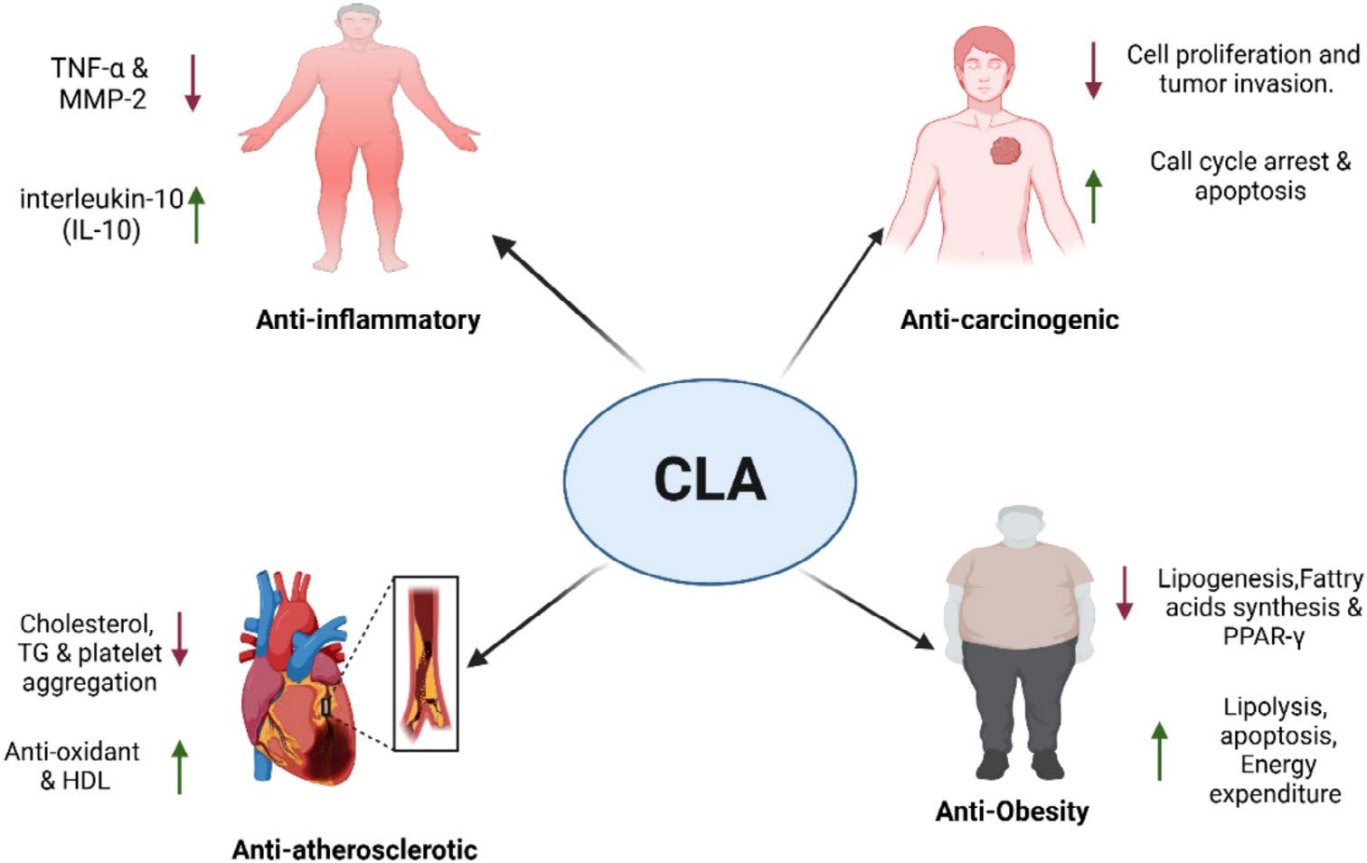
CLA exhibits various beneficial effects on biological systems.

### 6.2 Anti‐Obesity Effect

The primary focus has been on CLA's potential to inhibit obesity when used as a dietary supplement. CLA supplements of synthetic origin have been utilized in the majority of human intervention studies; the only consistently observed evidence pertains to their influence on weight loss and fat accumulation (Chooi et al. 2019). Over a duration of 12 weeks, the administration of 3.4 g of CLA daily reduces obesity indices in healthy overweight and obese adolescents without inducing any notable adverse effects (Mądry et al. 2020). While CLA supplements primarily influence low‐density lipoprotein (LDL) cholesterol levels, they beneficially affect the overall lipid profile (Derakhshande‐Rishehri et al. 2015). Confirmed mechanisms for the anti‐obesity action of CLA involve enhancing fatty acid oxidation, enhanced energy expenditure and lipolysis, and altering adipocyte metabolism, adipokines, and cytokines all—of which occur without modifying food intake or caloric intake (Onakpoya et al. 2012; Fuke and Nornberg 2017). The second suggested mechanism of action for CLA is through the peroxisome proliferator‐activated receptor (PPAR), which controls adipogenesis and lipid metabolism (Yuan et al. 2015; Wang et al. 2014; Wahli and Michalik 2012). Based on 118, the t10, c12‐CLA isomer suppresses adipogenesis and lipogenesis in adipocytes by altering PPAR‐γ target gene expression and modifying the transactivating function of PPAR‐γ by binding with SIRT1. Moreover, adipocyte formation is also impaired since it stabilizes β‐catenin, which subsequently interacts with PPAR‐γ. The interaction between them diminishes the transcriptional activity of PPAR‐γ and β‐catenin and thus makes it impossible for adipogenesis (Yeganeh et al. 2016).

### 6.3 Anti‐Inflammatory Effect

When stimulated by lipopolysaccharide, bovine immune cells could release less tumor necrosis factor if treated with the t10,c12‐CLA isomer (Perdomo et al. 2011). With the consumption of CLA‐enriched butter, it is possible to elevate concentrations of the anti‐inflammatory cytokine interleukin 10 (IL‐10), lower levels of tumor necrosis factor alpha (TNF‐α), IL‐2, IL‐8, matrix metalloproteinase‐2 (MMP‐2), matrix metallopeptidase‐9 (MMP‐9), etc. In young, healthy overweight individuals with signs of subclinical inflammation, this translates to lower systemic inflammatory mediators (Penedo et al. 2013). The c9,t11‐ and t10,c12‐CLA isomers can suppress the production of pro‐inflammatory cytokines such as TNF‐α, and they can also modulate the immunological microenvironment to support cell differentiation towards a more regulatory phenotype after antigen presentation (Viladomiu et al. 2016). The anti‐inflammatory action of CLA is caused by its capacity to stimulate PPAR (Murru et al. 2021).

### 6.4 Anti‐Carcinogenic Effect

It seems that redox changes, such as the ongoing formation of reactive oxygen species and reactive nitrogen species (ROS), are implicated in tumor promotion (Chaiswing and Oberley 2010). An incidence of lower mammary carcinogenesis has, in rats, been linked with higher serum levels of CLA (Polidori et al. 2018). Still, the very mechanisms by which CLA exhibits its anti‐carcinogenic actions remain undefined (Fuke and Nornberg 2017). Its anti‐inflammatory, antioxidant, and cell‐growth‐inhibiting effects are accountable for these benefits. Conversely, scientists in a mouse model of aggressive breast cancer discovered that CLA enhanced tumor growth and cell proliferation through modification of the mammary stromal environment, thereby promoting tumor growth (Flowers et al. 2010; Białek et al. 2015). The anti‐carcinogenic effects of CLA could be enhanced when it combines with polyunsaturated fatty acids (PUFAs) via the lipoxygenase (LOX) and cyclooxygenase (COX) pathways. The t10,c12‐CLA isomer might be able to suppress tumor growth by causing cell cycle arrest at the S phase; it is dose‐ and time‐dependent (Thuillier et al. 2013), according to in vivo evidence from mice and in vitro studies using the ovarian cancer cell line TOV‐21G.

### 6.5 Anti‐Atherosclerotic Effect

Atherosclerosis is one of the major causes of cardiovascular disease (CVD) and is described by the progressive inflammatory alteration in both the smooth and hard coronary arteries, which results in the accumulation of LDL cholesterol‐loaded particles under the endothelium layer (Albany et al. 2019). By reducing oxidative stress and mitochondrial impairment, CLA has been found to potentially decrease acrolein‐induced cardiotoxicity in rats (Aydın et al. 2018). Furthermore, spontaneously hypertensive rats have shown that CLA improves metabolic syndrome‐associated organ damage (Soto‐Rodríguez et al. 2011). Scientists have established that individuals whose diets contain CLA had substantially lower total cholesterol, low‐density lipoprotein cholesterol, and plasma triacylglycerol levels. The t10,c12‐CLA isomer could have an impact on the production of vasoactive molecules from adipose tissue, and this might account for its beneficial effects on blood pressure and adipocyte size in fa/fa Zucker rats by lowering the percentage of big adipocytes. Moreover, CLA supplement intake alters heart tissue fatty acid composition by decreasing cholesterol oxidation and malondialdehyde (MDA) content, which indicates that PUFA oxidation is largely suppressed (DeClercq et al. 2012; Białek et al. 2019). This may induce alterations in heart tissue function or structure.

## 7 Potential Risks of CLA in Humans and Animals

The predominant dangers are associated with the t10, c12‐CLA isomer, while the c9, t11‐CLA is presumably harmless for humanoid intake, but this necessitates verification (Yang et al. 2015). In humans, CLA supplements may induce insulin resistance or impair glucose absorption (Halade et al. 2010), particularly in those with diabetes or metabolic syndrome. Furthermore, CLA may adversely affect the liver, potentially resulting in hepatic steatosis (Bilal et al. 2015). It can also affect gut health, fertility, and vitamin A metabolism (Su et al. 2020; Zamora‐Zamora et al. 2017). In animals, the opposing effects of CLA predominantly encompass diminished milk fat production, lower poultry output, impaired egg quality, and modified fish performance (Zeitz et al. 2018; Anadón et al. 2010).

## 8 Dose‐Dependent Effects: When More Is Not Better

### 8.1 Low to Moderate Doses of CLA

When CLA is ingested at low doses, its advantageous effects on significant metabolic irregularities linked to metabolic syndrome (MetS) are preserved without adverse side effects (Martín‐González et al. 2020). The research conducted by Wan Shen et al. in 2013 showed that low concentrations of 10,12 CLA (0.03%, 0.1%, and 0.3%) enhanced the expression of uncoupling protein 1 (UCP1), a protein associated with lipolysis, in both subcutaneous and visceral adipose tissue (epididymal) in lean mice. Subsequently, it was found that a 0.1% dosage of 10,12 CLA might ease the alteration of white adipose tissue into brown‐like adipose tissue, even in mice subjected to a low‐fat diet (Shen et al. 2013). Shen et al. administered a significantly lower dosage of 10,12 CLA than Wendel et al. until now it effectively facilitated the transformation of both visceral and subcutaneous white adipose tissue into brown‐like adipose tissue. Furthermore, it exerted minimal adverse effects on liver weight (Shen et al. 2015). This investigation demonstrated no opposing effects of CLA on insulin signaling, despite its common association with insulin resistance (Pinto Júnior and Seraphim 2012). A low dose of CLA enhanced the rats' glucose metabolism. It facilitated the normalization of blood sugar, hepatic glucose, and insulin levels, counteracting the effects of the CAF diet. Furthermore, CLA decreased alanine (Petersen et al. 2017) and lactate (Rui 2014) concentrations in the liver, restoring them to normative levels.

### 8.2 High Doses of CLA

Meat and milk contain CLAs, which have been associated with various health issues (Scalerandi et al. 2014). Some isomers of CLA, evidence indicates, have been shown to cause insulin resistance in mice and humans and result in fat deposition within the livers of mice that are enlarged (Risérus et al. 2002; Tsuboyama‐Kasaoka et al. 2000). Bezan et al. (2018) established that male Wistar rats became more insulin resistant and accumulated hepatic fat following the administration of a high dose of CLA (3%), an equimixture of c9,t11‐CLA and t10,c12‐CLA. 10.1055, s‐0043‐118,348. Mice that were treated with CLA lowered milk fat secretion and raised liver weight because of hepatic steatosis, as evidenced in another similar study by Kadegowda et al. (2010).

## 9 Future Directions in CLA Research

CLA has been extensively deliberated for its possible health benefits, particularly with obesity and metabolism. Nonetheless, many unresolved inquiries and innovative therapeutic uses persist. Future research should aim to enhance our comprehension of CLA's molecular underpinnings, explore novel therapeutic applications, and integrate personalized nutrition recommendations. Although prior studies offer insights into the possible advantages of CLA in hominids, further investigation is essential to ascertain the ideal intake levels and to evaluate the short‐ and long‐term effects, including any potential opposing effects of specific CLA isomers, to guarantee their safety and efficacy. Future studies should emphasize dose management and investigate CLA's potential combination with traditional cancer therapies. Moreover, research trials ought to inspect the impact of numerous CLA isomers on cardiovascular metrics across diverse populations. Extended research studies are mandatory to assess whether the advantageous effects of CLA produced spontaneously in the humanoid gut differ from those of orally ingested CLA. CLA derived from natural dietary sources, particularly dairy fat, primarily consists of the c9,t11 isomer, associated with anti‐inflammatory properties. In contrast, supplements often offer a 50:50 mixture of c9,t11 and t10,c12 isomers, the latter potentially leading to detrimental metabolic consequences. Consequently, the source and isomer composition of CLA markedly affect its health consequences. CLA possesses nutritional and therapeutic efficacy for the management of obesity, dyslipidaemia, and inflammation‐related disorders. Nonetheless, its effectiveness and safety are contingent upon the isomer type, dosage, and individual metabolic reactions.

## 10 Conclusion

The function of CLA in obesity and metabolic disorders is intricate, presenting potential advantages that are mitigated by discrepancies in human studies. Although animal studies endorse its anti‐obesity and metabolic regulation properties, discrepancies in isomer composition, dosage, and individual metabolic responses lead to the variable results seen in clinical environments. CLA demonstrates promise in regulating lipid metabolism and inflammatory pathways; nonetheless, issues including insulin resistance, hepatic steatosis, and modified lipid profiles require careful consideration in its application. These findings highlight the necessity of transitioning from broad supplementation to individualized, dose‐optimized approaches. Future study must emphasize personalized nutrition by accounting for genetic predispositions and lifestyle factors, thereby integrating preclinical findings with clinical applicability to effectively utilize CLA's therapeutic potential.

## Author Contributions

**Magendran Rajendran:** conceptualization (equal), investigation (equal), methodology (equal), visualization (equal), writing – original draft (equal), writing – review and editing (equal). **Shanmugasundaram Palani:** investigation (equal). **Thakur Gurjeet Singh:** investigation (equal). **Brian Oliver:** investigation (equal). **Kaml Dua:** investigation (equal). **Vetriselvan Subramaniyan:** investigation (equal), methodology (equal), writing – review and editing (equal). **Kumaran Narayanan:** investigation (equal), methodology (equal), visualization (equal), writing – review and editing (equal).

## Conflicts of Interest

The authors received no specific funding for this work.

## Data Availability

No datasets were generated or analyzed during the current study.
